# Differences in the digestive enzyme activity, intestinal mucosa and microbial community in loach cultivated in two separate environments

**DOI:** 10.1186/s12866-018-1237-1

**Published:** 2018-09-10

**Authors:** Song Yang, Jie Du, Yuan-liang Duan, Qing Xiao, Ning-qiu Li, Qiang Lin, Liu-lan Zhao, Zong-jun Du, Jian Zhou, Jun Du

**Affiliations:** 1 College of Animal Science and Technology, Sichuan Agricultural University, Wenjiang, Chengdu, 611130 Sichuan China; 2 Key Laboratory of Fishery Drug Development, Ministry of Agriculture and Rural Affairs, Pearl River Fisheries Research Institute, Chinese Academy of Fishery Sciences, Guangzhou, 510380 China; 3 Fisheries Institute of Sichuan, Academy of Agricultural Science, Chengdu, 611731 Sichuan China

**Keywords:** Enzymatic activity, Intestinal microbiota, Loach, Paddy fields, Quantitative PCR

## Abstract

**Background:**

Fish culture in rice paddies can contribute to increasing yields of rice and surplus fish products. Environmental impacts and food-safety issues have become important topics in aquaculture, and organic foods currently were paid attention by researchers and industry practitioners. But the mechanism of differences in quality of Loach (*Paramisgurnus dabryanus*) reared in rice fields and ponds remains largely uncharacterized. In this study,digestive enzyme activity, intestinal mucosa cells and the gut microbial community of loach were determined under the two separate cultivation modes.

**Results:**

The levels of intestinal digestive enzyme activity of fish reared in the paddy-cultivated mode (PACM) were higher (*P* < 0.05) than those in the pond-cultivated mode (POCM). It was extremely significant (*P* < 0.01) for the activity of lipase in the liver, foregut and midgut, and for the activities of amylase and trypsin in the hindgut. Acid mucous cells in the loach foregut in PACM were fewer than in POCM (*P* < 0.01). In summer, the abundance of the Firmicutes, *Lactobacillus* spp., *Aeromonas hydrophila*, Enterobacteriaceae and *Streptococcus* spp. in loach intestinal mucosa in PACM was higher than in POCM. In fall, the abundance of total bacteria, the Bacteroidetes, *Bifidobacterium* and Enterobacteriaceae in the intestinal mucosa in PACM was likewise higher than in POCM. These differences were significant (*P* < 0.05 or *P* < 0.01) between loach in the two separate culture modes for all microorganisms except for *A. hydrophila* and *Streptococcus* spp. In addition, quantitative PCR assays showed that some microorganisms presented consistently similar abundances in the gut as in the culture water.

**Conclusions:**

These results showed some enzymatic activities involved in digestion in liver and intestine of loach in PACM were higher than those in POCM, as using digestive enzyme analysis and histological observation of intestinal sections. These findings suggest most of the microorganisms examined in the gut mucosa of loach in the two culture modes significantly differed in abundance between summer and fall. However, some pathogenic bacteria in the gut, particularly *A. hydrophila*, presented lower abundance in PACM in fall, yet did not differ in abundance between loach in the two cultivation modes*.*

## Background

The intestinal tract of vertebrates plays a critical role in absorbing nutrients and protecting the host from pathogens [1, 2]. It is well recognized that the bacterial flora in the fish gut has beneficial effects for the host [3]. The intestinal microbiota form a symbiotic relationship with the host and are crucial for the host in many ways, including balancing the immune response, promoting digestion and mediating host physiology [4]. But the stability of the relationship, particularly in the intestine where some microorganisms help to protect against pathogens, was easily affected by numerous factors, such as water temperature, pH, and the diet and species of the fish [5]. Numerous comparative studies of the microbiota have examined fish from various geographical areas [6], and the composition of the gut microbiota has been examined in wild and reared salmonids, croaker and yellowtail [7]. The absorptive cells and goblet cells are primary types of mucosal epithelial cells [8]. Goblet cells in the gastrointestinal tract can secrete neutral and acid mucopolysaccharides (the former stain red, and the latter stain blue), and thus these cells display various colors ranging between blue and purple-red. For example, the purple-blue cells contain mixed polysaccharides, but the content of acid mucopolysaccharides present more. Goblet cells secrete mucus that provides a mechanical and chemical barrier with an immune function in the intestinal wall [9–11]. Together, these contribute to the host’s absorption of nutrients and protect it against pathogens. Thus, increasing evidences indicate that the normal structure and function of the intestinal tract can strengthen intestinal digestion and absorption process [12], and also can enhance the immune system to protect the host from invasion via the external environment.

Fish are a global significant commodity based on their potential to improve food security and human nutrition [13]. The role of aquaculture is not only to ensure supplies of protein but also to provide healthy food sources. Environmental impacts and food-safety issues have become important topics in aquaculture, with organic sources of food receiving attention from both researchers and industry worldwide [14]. However, rapid expansion of intensive aquaculture systems frequently has negative consequences, such as outbreaks of disease among the cultured organisms, the misapplication of drug treatments, and adverse impacts on the environment [15]. Integrated agri-aquaculture systems are using ecological approaches for the production of various crops and animal species [16]. To improve the quality of production while protecting the environment, microecology technology may be used in these aquaculture systems. This mainly involves the use of probiotics in fish feeds or changes of the culture environment to improve the health and gut microbiota of the fish. Fish culture in paddy fields represents a classic integrated aquaculture system, with major and minor components – namely, rice planting combined with fish breeding in the system [17]. Historical practice has proven that culturing fish in paddy fields has many advantages, such as a lower level of investment and increased yields of the rice and aquatic animal product [18].

The objectives of the present study were to: 1) analyze the digestive enzyme activity of lipase, amylase and trypsin in the liver, foregut, midgut and hindgut of loach in the two culture modes, namely paddy-cultivated mode (PACM) and pond-cultivated mode (POCM); 2) observe the histological structure and distribution of intestinal mucous cells (based on Alcian blue– periodic acid-Schiff staining) in loach under the two culture modes; and 3) compare the abundance and dynamic state of certain intestinal microorganisms (that may have a good or bad effect on the host) in loach during different seasons and under the two cultivation modes, using quantitative PCR (Q-PCR) assays. Thus, we designed an experiment to reveal the structure of the intestinal microbiota in loach reared in the two separate environments, and to assess whether rearing loach in paddy fields better than in ponds.

## Methods

### Fish and sampling

The loach, *Paramisgurnus dabryanus,* were collected randomly from three paddy fields (N: 29°22′59″, E: 105°11′11″, Longchang County, Neijiang City, Sichuang Province, China) and three ponds (N: 29°22′15″, E: 105°11′2″, Longchang County, Neijiang City, Sichuang Province, China). In this study, the fish were firstly cultivated in two different rearing environments (paddy field and pond modes). They were fed with the same diets from May 5 to November 5 (2015) in three paddy fields and three ponds. The sizes of paddies and ponds were almost 666 m^2^. They were bred at different stocking densities (a hundred thousand for pond and ten thousand for paddy) and same management. The fish were fed three times per day with same commercial feed at feed rate of 3~ 5% body weight day^− 1^. The fish were firstly sampled in the summer (August 2015, water temperature 21 °C, fish fries had been farmed for three months) and were secondly sampled in the fall (November 2015, water temperature 20 °C, the weight of loach from paddy fields and the ponds be 46.03 ± 5.08 g and 48.69 ± 5.32 g, respectively). There was only 1 °C of difference in the water temperature between summer and fall, it owing to the water flows in the mountain stream and the specific heat capacity of water is larger. Fish samples were collected for analysis in the August (summer season) and November (fall season) during the feeding trial. The samples, only used for intestinal microbiota analysis were sampled in summer and fall, while the fish were sampled in fall, used for the enzyme activities and morphology. Ten healthy individuals were randomly sampled from each farming pond and each time.

In the study, for the digestive enzymatic activities and intestinal mucous cells analysis experiments, the fish were carried to the laboratory in oxygen filling bags within 3 h. Besides the samples were directly collected in fish farm for gut microbial analysis, and then the samples were also carried to the laboratory in ice within 3 h. The fish were euthanized by overdose of MS-222 (Sigma, Germany) before dissection [19]. The surface of the fish was rinsed with sterile distilled water and 70% ethanol to reduce contamination, before dissection with flamed sterile scissors. The intestine tracts were divided into the foregut, midgut and hindgut. In these studies, foregut was defined as the first section of intestine from esophagus to the distal end of the swelling of the intestine. The narrow middle section was defined as the midgut, and the larger-diameter section following this to just prior to the anus was defined as hindgut. Then they were frozen at − 80 °C for analysis of the digestive enzymatic activities analysis. Segments of 0.5 cm of the foregut, midgut and hindgut were collected from fish and fixed in fresh bouin’s solution at room temperature for intestinal mucous cells analysis by light microscopy (LM). And the intestine tracts were removed aseptically from their abdominal cavity and the content of intestine was squeezed out and separately stored. Thereafter, the mucosa in the epithelial intestinal of the loach was collected by blades, respectively. About 0.3 g samples of content and mucosa were used for bacterial extraction. Meanwhile, the water was also sampled at an approximate depth of 35 cm from five sites in the each paddy or pond, pooled it together and 300 ml water was stored for centrifuging. The pellet was collected after centrifuged at 13000×g for 20 min at 4 °C. All of the mucosa, content and water samples were collected under sterile conditions and stored at − 80 °C for microbiota analysis by Q-PCR.

### Intestinal digestive enzymatic activities

Ten healthy individuals were randomly sampled from paddy fields and ponds, respectively. The number of individuals is 20 in this experiment. Intestine samples were washed with cold deionized water to remove most of the mucus, and the intestine was ground to pulp with cold sodium phosphate buffer (0.1 M, pH 7.0, 4 °C) by ratio of 1:9 (m/v). Then, the homogenate was centrifuged (3-18 K, Sigma®, Germany) at 4 °C at 3000×g for 10 min. The supernatant containing enzymes was stored at − 80 °C prior to the analysis. The lipase, amylase, trypsin and total protein were detected using assay Kits (Lipase assay kit, Amylase assay kit, Trypsin assay kit and Total protein quantitative assay kit) purchased from Nanjing Jiancheng, Bioengineering Institute, China. The specific procedures were reference kit’s instructions, and the protein concentration was used to normalize the enzymatic activity.

### Intestinal histological observation

Ten healthy individuals were randomly sampled from paddy fields and ponds, respectively. The total number of individuals is 20 in this experiment. Fixed samples were wrapped in gauze and rinsed in running water for 12 h, dehydrated in graded ethanol solution and embedded. Sections were cut at 6 μm serially using a rotary microtome. The sections were stained with alcian blue and periodic acid schiff (AB-PAS) and photo-documented using a low power light invert microscope (Nikon, Japan), measured with Photoshop CS4. In different histological sections, the numbers of mucous cells in multiple micrographs from each intestinal region of ten fish in each group were measured, and twelve micrographs from the foregut, midgut and hindgut of intestinal samples were chosen.

### Q-PCR quantification of microbiota

Ten healthy individuals were randomly sampled from two seasons and two modes (summer and fall; paddy fields and ponds), respectively. The total number of individuals is 40 in this experiment. Firstly, the total bacterial DNA of the samples was extracted using TIANamp Bacteria DNA Kit (TIANGEN, China). Then, in order to estimate the abundance of beneficial bacteria and harmful bacterica in loach gut, a gold method and citation was referenced [21]. Basing on the method discribed by Sun H. (2016), some instructive detection method and guidances were used to check the biomass, such as Q-PCR. The part primers for Q-PCR of microbiota were also listed in Table 1, including references. The reaction system components and reaction procedures are summarized in Table 2.

**Table 1:** Primer information and standard curves of microflora for Q-PCR

Bacterial species	Primer sequence (5 → 3)	Regression curve/Tm	Reference
Total bacteria	F: CGGYCCAGACTCCTACGGG	y = 14.354–0.2607X	[20]
	R: TTACCGCGGCTGCTGGCAC	R^2^ = 0.997 Tm = 59.5 °C	
Firmicutes	F: GGAGYATGTGGTTTAATTCGAAGCA	y = 13.073–0.2985X	[21]
	R: AGCTGACGACAACCATGCAC	R^2^ = 0.998 Tm = 60.5 °C	
Bacteroidetes	F: GGARCATGTGGTTTAATTCGATGAT	y = 14.390–0.2807X	[21]
	R: AGCTGACGACAACCATGCAG	R^2^ = 0.998 Tm = 58 °C	
*Bifidobacterium*	F: TCGCGTCYGGTGTGAAAG	y = 13.837–0.2045X	[21]
	R: CCACATCCAGCRTCCAC	R^2^ = 0.998 Tm = 61.5 °C	
*Enterococcus* spp.	F: CCCTTATTGTTAGTTGCCATCATT	y = 14.356–0.2542X	[21]
	R: ACTCGTTGTACTTCCCATTGT	R^2^ = 0.998 Tm = 52 °C	
*Lactobacillus* spp.	F: AGCAGTAGGGAATCTTCCA	y = 14.899–0.2863X	[22]
	R: CACCGCTACACATGGAG	R^2^ = 0.999 Tm = 58 °C	
*A*. *hydrophila*	F: GAAAGGTTGATGCCTAATACGTA	y = 13.903–0.2050×	
	R: CGTGCTGGCAACAAAGGACAG	R^2^ = 0.991 Tm = 53.5 °C	
Enterobacteriaceae	F: CATTGACGTTACCCGCAGAAGAAGC	y = 13.870–0.1927X	[23]
	R: CTCTACGAGACTCAAGCTTGC	R^2^ = 0.995 Tm = 53 °C	
*Streptococcus* spp.	F: AGAGTTTGATCCTGGCTCAG	y = 14.086–0.2630X	[24]
	R: GTTAGCCGTCCCTTTCTGG	R^2^ = 0.996 Tm = 59.5 °C	

Note: “X” is representative the value of “Ct” of PCR, “C” representative the “Cycle”, “t” representative the “threshold”. And “y” is representative the Log10 DNA gene copies quantification data

**Table 2:** The reaction system components and procedures of Q-PCR

components	SYBR® Premix Ex Taq™ II	forward and reverse primer (10 μM)	template DNA	sterile deionized water	total
volume	12.5 μl	2 μl	1 μl	9.5 μl	25 μl
reaction procedures	predenaturation	40 cycles			extension
		denaturation	annealing	extension	
Temperature (°C)	95	94	optimal temperature	72	72
Time (s)	60	15	30	30	10

The triplicate tenfold serial dilutions of the plasmid DNA were used to built the standard curves. Based on the standard curves, copy numbers of the target bacterial phylum or genus in samples were calculated. The method described by Sun H. et al. (2016) [21] was performed to built the standard curves of quantification of microbial by quantitative PCR. The limit of detection of Q-PCR was 5 × 10^1^.

### Statistical analysis

The raw data were firstly imputed into Excel to setup database because the two data types could transform between Excel and SPSS software. Statistical analysis was performed using a one-way ANOVA in SPSS 19.0 software (SPSS Inc., Chicago, Illinois, USA). Data were presented as mean ± SD (standard deviation). The difference was evaluated by one-way ANOVA and at level of *P* < 0.05 or *P* < 0.01 for significance.

## Results

### Digestive enzyme activities

Enzymatic activity in the loach intestine in autumn is summarized in Table 3. The levels of digestive enzyme activity were significantly higher (*P* < 0.01) in the liver and foregut than in the midgut and hindgut for fish in both culture modes. In PACM, the activities of lipase and amylase were highest in foregut and lowest in hindgut; the activity of trypsin was highest in liver and lowest in hindgut. In POCM, the activities of lipase, amylase and trypsin presented a similar trend in the different tissues, with the activities of these three digestive enzymes always highest in liver and foregut and lowest in the hindgut. The activity of lipase in liver, foregut and midgut was significantly higher in PACM than in POCM (*P* < 0.01); in contrast, the activities of amylase and trypsin were lower in just the liver and foregut but higher in the hindgut in PACM as compared with POCM (*P* < 0.01).

**Table 3:** The digestive enzyme activities of loach in different culture modes

Cultivation mode	Tissue	Enzyme activity		
		lipase (U g^−1^prot)	amylase (U mg^− 1^prot)	Trypsin (U mg^− 1^prot)
PACM	Liver	806.49 ± 31.00^A^**	2.16 ± 0.09^Aa^**	3576.48 ± 23.62^A^**
	Foregut	878.75 ± 7.14^B^**	2.32 ± 0.09^Ab^**	3471.50 ± 6.27^B^**
	Midgut	388.33 ± 5.63^C^**	1.43 ± 0.06^B^*	1139.15 ± 10.86^C^
	hindgut	139.26 ± 3.88^D^*	0.59 ± 0.03^C^**	144.04 ± 5.18^D^**
POCM	Liver	606.41 ± 20.85^A^	2.68 ± 0.06^Aa^	3843.50 ± 15.04^Aa^
	Foregut	656.30 ± 9.88^B^	2.71 ± 0.09^Aa^	3889.16 ± 31.27^Ab^
	Midgut	229.12 ± 7.91^C^	1.29 ± 0.03^B^	1138.77 ± 18.26^B^
	hindgut	124.79 ± 5.45^D^	0.43 ± 0.03^C^	115.86 ± 4.22^C^

Note: Values are as mean ± standard deviation, *n* = 20. The significance analysis used in same enzyme and cultivation mode between different tissues with the letter. The capital superscript letters in the some row represent the significant difference (*P* < 0.01); the superscript lowercase letters represent difference (*P* < 0.05); and there are no differences with same letter (*P* > 0.05). The significance analysis used in same tissue and enzyme between different cultivation modes with *. ** represent the significant difference (*P* < 0.01), *represent difference (*P* < 0.05)

### Intestinal mucous cells

**Fig. 1:**
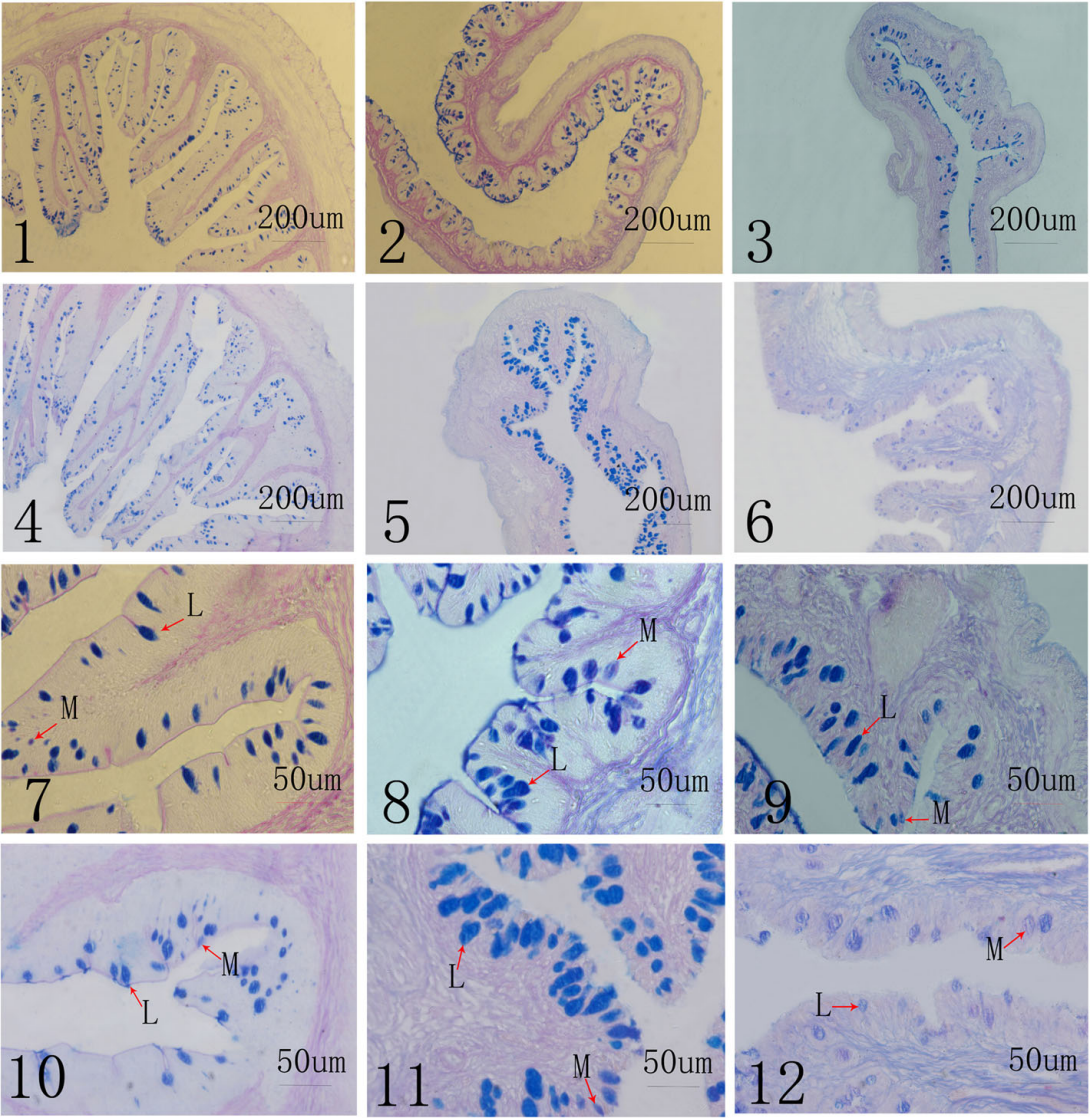
AB-PAS staining inverted microscopy micrographs (different parts of intestinal samples arbitrarily chosen as examples, 1-6: 100×; 7-12: 400×). 1, 2, 3, 7, 8 and 9 is the samples from paddy fields, and 4, 5, 6, 10, 11 and 12 is the samples from ponds. 1, 4, 7, 10: foregut; 2, 5, 8, 11: midgut; 3, 6, 9, 12: hindgut. L represent the acid mucous cells, M represent the partial acid mucous cells

A summary of the light-microscope observations of the intestinal-tissue structures are presented in Table 4. In general, the numbers of mucous cells gradually decreased from the foregut to hindgut in the intestine of loach not only in PACM but also in POCM (*P* < 0.01). In PACM, the numbers of acid mucous cells in the foregut, midgut and hindgut were significantly higher than the numbers of purple-blue cells (*P* < 0.01). The numbers of acid mucous cells showed a similar increasing trend from foregut to hindgut in POCM (*P* < 0.01). The numbers of mucous cells mostly presented significant differences (*P* < 0.01) among same the intestinal segments of loach in PACM as compared with in POCM, including the acid mucus cells and purple-blue cells. However, the difference was not significant between the two cultivation modes in spite of higher numbers of acid mucous cells in the hindgut in PACM. In addition, the size of the mucous cells tended to be bigger in the midgut than in the foregut (Fig. 1).

**Table 4:** The number of the mucous cells in different culture modes

mucous cells (view^−1^)	PACM			POCM		
	foregut	midgut	hindgut	foregut	midgut	hindgut
acid	2839.00 ± 12.29^A^**	954.00 ± 5.57^B^**	223.67 ± 7.23^C^	3553.67 ± 9.50^A^	904.33 ± 5.51^B^	214.00 ± 2.65^C^
partial acid	108.33 ± 3.21^A^**	57.00 ± 2.00^B^**	20.67 ± 2.08^C^**	186.00 ± 6.00^A^	73.33 ± 4.04^B^	33.33 ± 1.53^C^

Note: Values are as mean ± standard deviation, *n* = 20. A view stands for a whole transverse section of the intestinal. In the same cultivation modes, the differences of capital letters represent the significant difference (*P* < 0.01) among different intestinal segments. In the different cultivation modes, **represent the significant difference (*P* < 0.01) among same intestinal segments

### Q-PCR evaluations of the microbiota

**Fig. 2:**
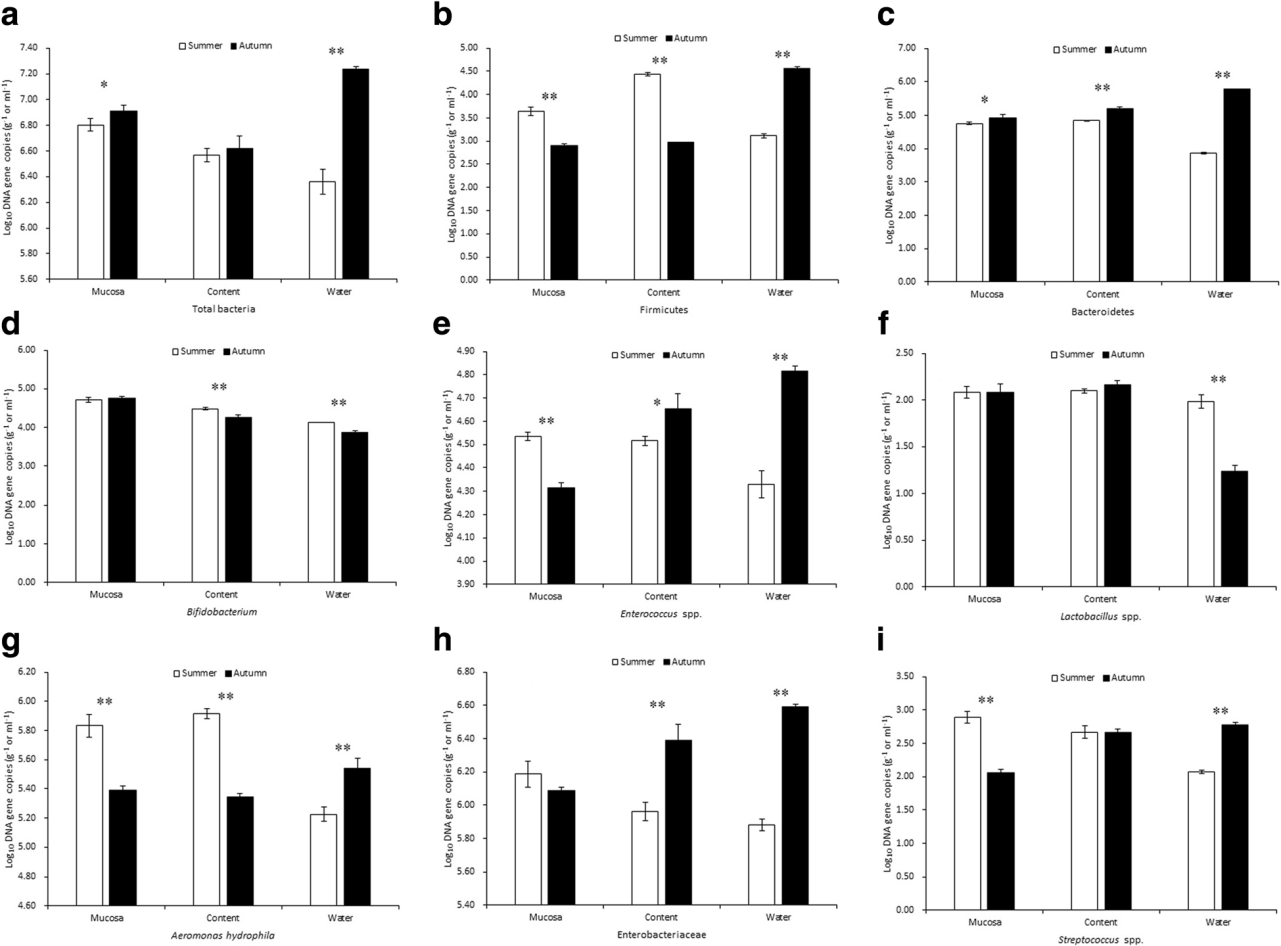
Effects of different seasons on functional bacteria (paddy cultivation modes). Note: **a**–**i** represent Log_10_ DNA gene copies of total bacteria, Firmicutes, Bacteroidetes, *Bifidobacterium*, *Enterococcus* spp., *Lactobacillus* spp., *A*. *hydrophila*, Enterobacteriaceae and *Streptococcus* spp. in the mucosa, content and water, respectively. Log_10_ DNA gene copies quantification data were normalized to the standard curve lines and presented with the means ± standard deviation (*n* = 20). * indicate *P* < 0.05, ** indicate *P* < 0.01

In PACM, the loach displayed a greater abundance of total bacteria, the Firmicutes, Bacteroidetes, *Bifidobacterium*, *Enterococcus* spp., *Lactobacillus* spp., *Streptococcus* spp., Enterobacteriaceae and *Aeromonas hydrophila* in the intestinal contents and mucosa than found in the rearing water in the summer samples as compared with the autumn samples, except the difference was not significant for *Lactobacillus* spp. (Table 5). Considerable variation in the fish mucosa was detected in different seasons, including different abundances of total bacteria, the Bacteroidetes and *Bifidobacterium*. In summer, the abundances of total bacteria, the Firmicutes, the Bacteroidetes, *Bifidobacterium*, and some pathogenic bacteria (*A*. *hydrophila*) were remarkably higher in the mucosa than in the water, and the difference was significant (*P* < 0.01) for the Firmicutes, *Enterococcus* spp., *A*. *hydrophila* and *Streptococcus* spp. Moreover, substantial amplification difference was observed in the summer contents, such as the abundance of total bacteria, the Bacteroidetes, *Enterococcus* spp., *Lactobacillus* spp. and Enterobacteriaceae. The abundance of *Enterococcus* spp. also differed remarkably between seasons (*P* < 0.05). In contrast, the abundances of most bacteria presented a reverse trend in the fall, becoming greater in the culture water than in the intestinal contents and mucosa (*P* < 0.05 or *P* < 0.01). Interestingly, the abundances of all the bacterial groups, except for *Bifidobacterium* and *Lactobacillus* spp., were significantly higher in the culture water than in the fish gut (*P* < 0.01) (Fig. 2).

**Table 5:** Log_10_ DNA gene copies of bacteria in different tissues (PACM)

	summer			Fall		
	mucosa	content	water	mucosa	content	water
Total bacteria	6.80 ± 0.05^A^	6.59 ± 0.05^Ba^	6.36 ± 0.10^Bb^	6.91 ± 0.05^A^	6.62 ± 0.09^B^	7.23 ± 0.02^C^
Firmicutes	3.64 ± 0.09^A^	4.44 ± 0.04^B^	3.11 ± 0.05^C^	2.90 ± 0.05^Aa^	2.97 ± 0.01^Ab^	4.56 ± 0.04^B^
Bacteroidetes	4.75 ± 0.04^Aa^	4.84 ± 0.02^Ab^	3.87 ± 0.04^B^	4.93 ± 0.10^A^	5.20 ± 0.04^B^	5.77 ± 0.01^C^
*Bifidobacterium*	4.72 ± 0.07^A^	4.49 ± 0.03^B^	4.14 ± 0.00^C^	4.76 ± 0.05^A^	4.27 ± 0.06^B^	3.87 ± 0.03^C^
*Enterococcus* spp.	4.54 ± 0.02^A^	4.52 ± 0.02^A^	4.33 ± 0.06^B^	4.31 ± 0.02^A^	4.65 ± 0.07^B^	4.82 ± 0.02^C^
*Lactobacillus* spp.	2.08 ± 0.06	2.10 ± 0.02	1.98 ± 0.07	2.08 ± 0.09 ^A^	2.16 ± 0.05 ^A^	1.24 ± 0.06^B^
*A*. *hydrophila*	5.83 ± 0.08^A^	5.92 ± 0.03^A^	5.23 ± 0.05^B^	5.39 ± 0.03^A^	5.34 ± 0.02^A^	5.54 ± 0.07^B^
Enterobacteriaceae	6.19 ± 0.08^A^	5.96 ± 0.06^B^	5.88 ± 0.03^B^	6.09 ± 0.02^A^	6.39 ± 0.09^B^	6.59 ± 0.02^C^
*Streptococcus* spp.	2.89 ± 0.09^Aa^	2.67 ± 0.09^Ab^	2.07 ± 0.02^B^	2.06 ± 0.05^A^	2.67 ± 0.04^Ba^	2.78 ± 0.03^Bb^

Note: Log_10_ DNA gene copies quantification data were normalized to the standard curve lines and presented with the means ± standard deviation (*n* = 20). In the same season, the capital superscript letters in the some row represent the significant difference (*P* < 0.01); the superscript lowercase letters represent difference (*P* < 0.05); and there are no differences with same letter (*P >* 0.05)

**Fig. 3:**
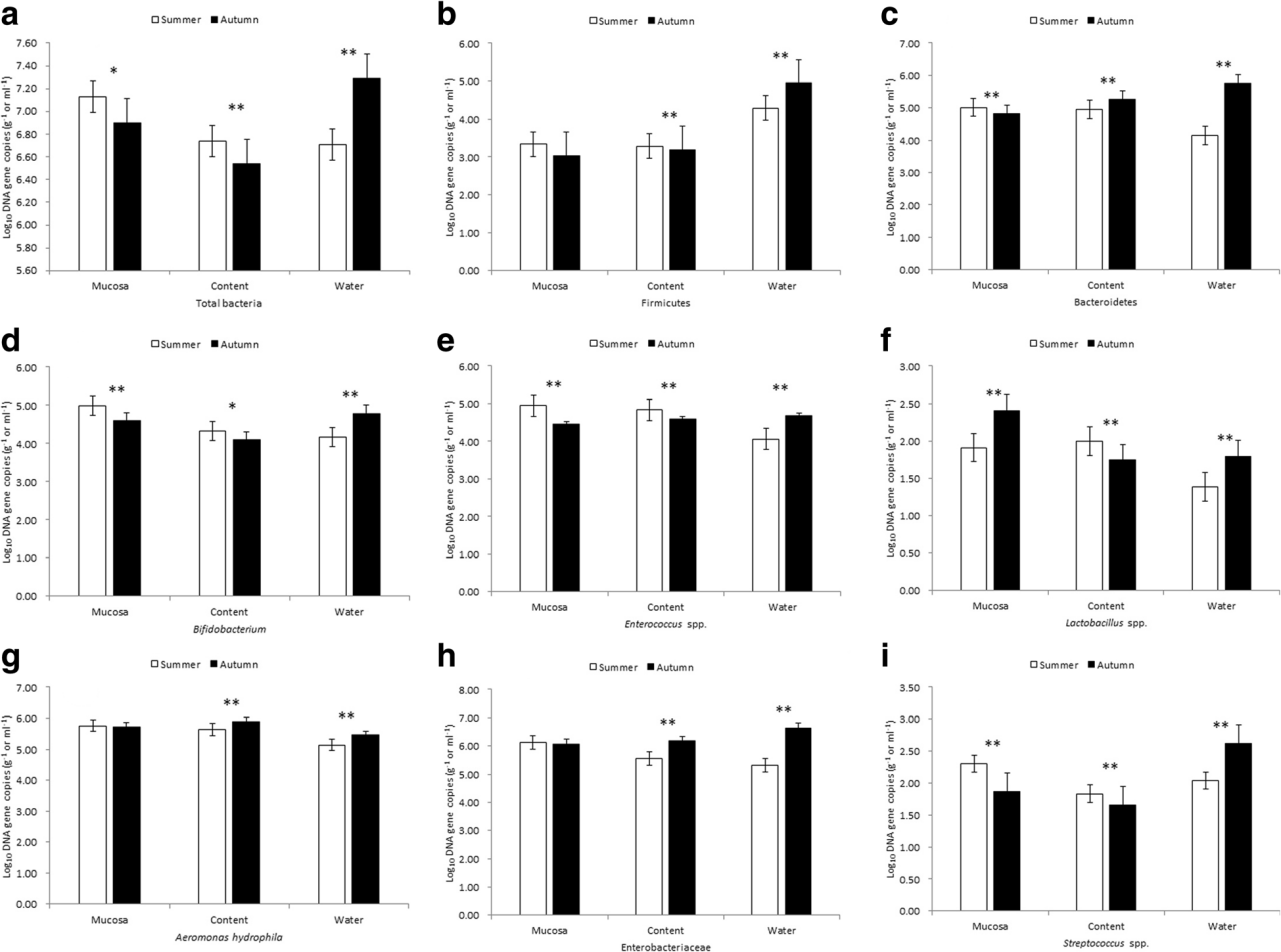
Effects of different seasons on functional bacteria (pond cultivation modes). Note: **a**–**i** Log_10_ represent DNA gene copies of total bacteria, Firmicutes, Bacteroidetes, *Bifidobacterium*, *Enterococcus* spp., *Lactobacillus* spp., *A*. *hydrophila*, Enterobacteriaceae and *Streptococcus* spp. in the mucosa, content and water, respectively. Log_10_ DNA gene copies quantification data were normalized to the standard curve lines and presented with the means ± standard deviation (*n* = 20). * indicate *P* < 0.05, ** indicate *P* < 0.01

In POCM, the abundances of the Firmicutes in the intestinal contents and mucosa, and *Streptococcus* spp. in the intestinal contents, were lower than found in the water in both summer and fall, while the other bacterial groups presented a higher abundance in summer. *Lactobacillus* spp. in the mucosa, and *A*. *hydrophila* in the intestinal contents and mucosa, maintained higher abundances than found in the water in the autumn samples, and other bacterial groups maintained lower abundances in the loach microflora than found in the water (Table 6). Considerable amounts of bacteria were detected in the loach mucosa in both seasons, and only the abundance of *Lactobacillus* spp. increased gradually from summer to fall. With dropping water temperatures, the abundance of total bacteria decreased in the intestinal contents and mucosa, and also in the water (*P* < 0.05); similar trends were observed for the abundances of the Bacteroidetes, *Bifidobacterium*, *Enterococcus* spp., *Lactobacillus* spp. and *Streptococcus* spp. between summer and fall (*P* < 0.01). Moreover, some of the dominant bacteria still maintained high abundance in the intestinal contents or mucosa than in the water in the fall, specifically *A*. *hydrophila* and Enterobacteriaceae (*P* < 0.01) (Table 6). Notably, the abundances of all bacterial groups in the fall were significantly higher in the culture water than in the fish microflora (*P* < 0.01) (Fig. 3).

**Table 6:** Log_10_ DNA gene copies of bacteria in different tissues (POCM)

	summer			Fall		
	mucosa	content	water	mucosa	content	water
Total bacteria	7.13 ± 0.07^A^	6.74 ± 0.06^Ba^	6.71 ± 0.05^Ba^	6.90 ± 0.08^A^	6.54 ± 0.02^B^	7.29 ± 0.09^C^
Firmicutes	3.34 ± 0.01^Aa^	3.29 ± 0.08^Aa^	4.29 ± 0.04^B^	3.04 ± 0.07^Aa^	3.19 ± 0.06^Ab^	4.95 ± 0.01^B^
Bacteroidetes	5.02 ± 0.01^Aa^	4.95 ± 0.04^Aa^	4.14 ± 0.05^B^	4.83 ± 0.03^A^	5.26 ± 0.02^B^	5.75 ± 0.00^C^
*Bifidobacterium*	4.99 ± 0.08^A^	4.33 ± 0.07^Ba^	4.17 ± 0.02^Bb^	4.59 ± 0.05^A^	4.09 ± 0.08^B^	4.79 ± 0.05^C^
*Enterococcus* spp.	4.95 ± 0.01^Aa^	4.83 ± 0.04^Ab^	4.06 ± 0.06^B^	4.46 ± 0.03^A^	4.59 ± 0.03^B^	4.69 ± 0.01^C^
*Lactobacillus* spp.	1.91 ± 0.07^Aa^	2.00 ± 0.06^Aa^	1.39 ± 0.09^B^	2.41 ± 0.03^A^	1.74 ± 0.04^Ba^	1.80 ± 0.06^Ba^
*A*. *hydrophila*	5.76 ± 0.03^A^	5.64 ± 0.04^B^	5.14 ± 0.03^C^	5.73 ± 0.10^Aa^	5.89 ± 0.06^Aa^	5.46 ± 0.07^B^
Enterobacteriaceae	6.12 ± 0.04^A^	5.55 ± 0.04^B^	5.30 ± 0.06^C^	6.05 ± 0.07^Aa^	6.17 ± 0.02^Ab^	6.64 ± 0.01^B^
*Streptococcus* spp.	2.30 ± 0.07^A^	1.83 ± 0.03^B^	2.04 ± 0.07^C^	1.87 ± 0.04^A^	1.65 ± 0.04^B^	2.62 ± 0.09^C^

Note: Log_10_ DNA gene copies quantification data were normalized to the standard curve lines and presented with the means ± standard deviation (*n* = 20). In the same season, the capital superscript letters in the some row represent the significant difference (*P* < 0.01); the superscript lowercase letters represent difference (*P* < 0.05); and there are no differences with same letter (*P >* 0.05)

**Fig. 4:**
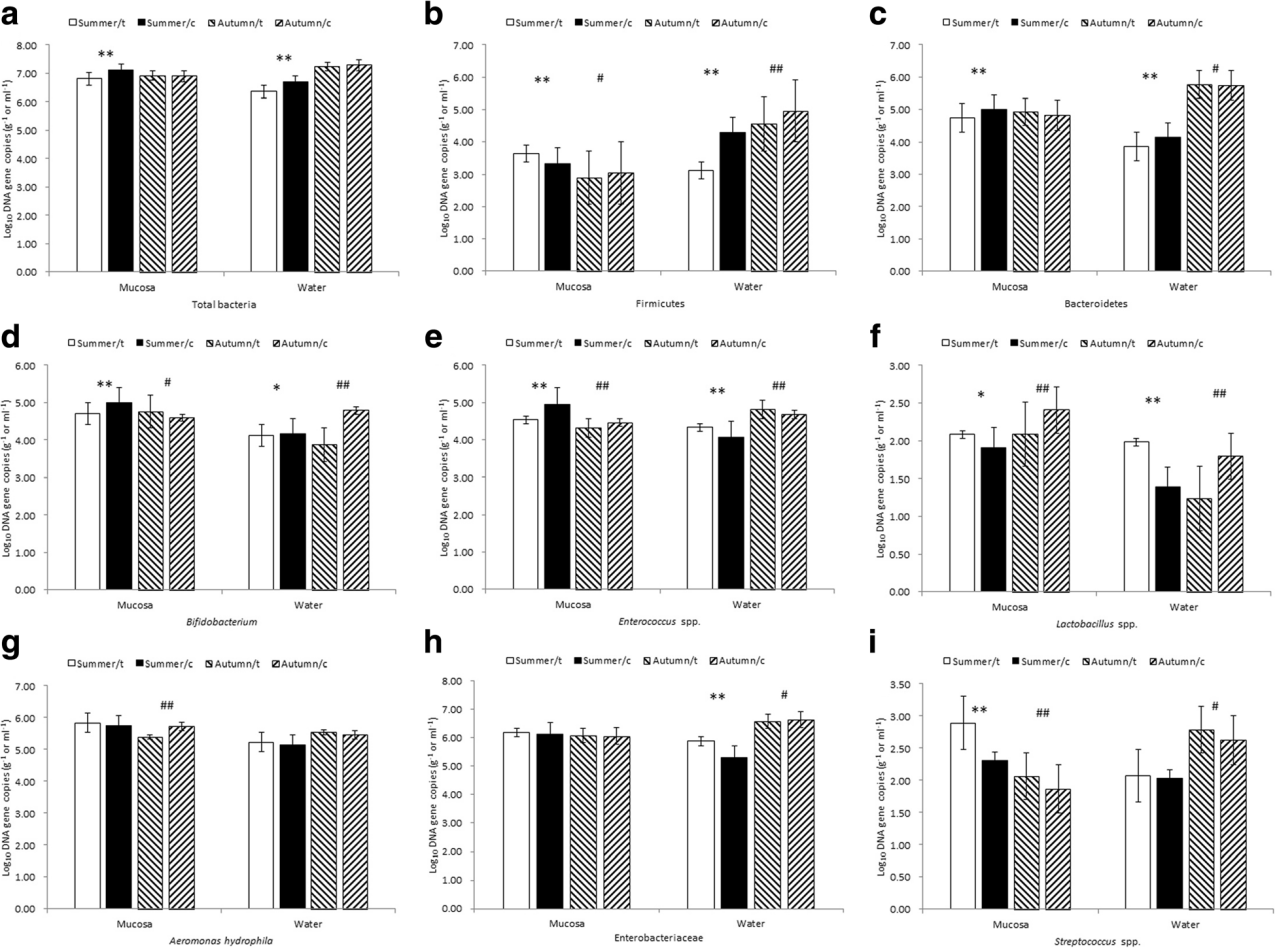
Effects of different cultivations modes on functional bacteria. Note: **a**–**i** Log_10_ represent DNA gene copies of total bacteria, Firmicutes, Bacteroidetes, *Bifidobacterium*, *Enterococcus* spp., *Lactobacillus* spp., *A*. *hydrophila*, Enterobacteriaceae and *Streptococcus* spp. in the mucosa and water, respectively. Log_10_ DNA gene copies quantification data were normalized to the standard curve lines and presented with the means ± standard deviation (*n* = 40). Summer/t and Summer/c represent the paddy and pond samples in summer, respectively; Autumn/t and Autumn/c represent the paddy and pond samples in autumn, respectively. In the summer samples, * indicate *P* < 0.05, ** indicate *P* < 0.01; in the autumn samples, # indicate *P* < 0.05, ## indicate *P* < 0.01

In summer, the abundance of the Firmicutes, *Lactobacillus* spp., *A*. *hydrophila*, Enterobacteriaceae and *Streptococcus* spp. in the loach mucosa was higher in PACM than in POCM, and the difference was significant (*P* < 0.05 or *P* < 0.01) except for *A*. *hydrophila* and Enterobacteriaceae. Moreover, the abundance of *Enterococcus* spp., *Lactobacillus* spp., *A*. *hydrophila*, Enterobacteriaceae and *Streptococcus* spp. was higher in the culture water in PACM than in POCM; this difference was significant (*P* < 0.05 or *P* < 0.01) for all microbiota except for *A*. *hydrophila* and Enterobacteriaceae. In the fall, the abundances of total bacteria, the Bacteroidetes, *Bifidobacterium* and Enterobacteriaceae in the mucosa were higher in PACM than in POCM, reaching significantly different levels (*P* < 0.05 or *P* < 0.01) except for *A*. *hydrophila* and *Streptococcus* spp. Furthermore, the abundances of the Bacteroidetes, *Enterococcus* spp., *A*. *hydrophila* and *Streptococcus* spp. in the culture water in PACM were higher than those in POCM, and these differences were significant (*P* < 0.05 or *P* < 0.01) except for the total bacteria and *A*. *hydrophila* (Fig. 4).

## Discussion

Loaches are stomachless fish, and the anterior intestinal swelling (foregut) serves as an ichthyic stomach. The histological analysis showed that the intestinal tract of loach could be generally divided into mucosa, submucosa, and muscular coats and serosa from the interior to exterior [25]. In this study, a high content of digestive enzyme activity occurred in the liver and foregut (Table 3). Thus, the liver and foregut are assumed to play an important role in digestion and nutrient absorption in this species, which was confirmed by the large numbers of the different types of mucous cells (Table 4) and observations of the mucous-cell morphology in the foregut (Fig. 1). Previous studies focused on the digestive enzymes in young fish or comparisons of different developmental stages of fish. Moyano [26] studied the activities of digestive enzymes during larval development in gilthead seabream; the results revealed that enzymatic activities increased in relation to fish development, and exogenous food had more of a qualitative than quantitative role in the secretion of digestive enzymes. Kuz’mina [27] studied the influence of fish age on the activities of digestive enzymes in several freshwater teleosts and found that total enzymatic activity in particular cases increased with age. In this study, the levels of digestive enzyme activity significantly differed between loach in the two cultivation modes. Loach reared in paddy fields may be more dependent on the environment; while they can prey on some live, foods they might also unavoidably suffer from starvation due to the environment. Kolkovski [28] discovered that live food organisms could promote enzymatic activity in larval and juvenile fish by ‘donating’ their digestive enzymes; not all the loaches cultivated in ponds depended on the commercial feed offered. Previous research revealed that the activities of digestive enzymes were directly affected by the food [29] and also changed in the fish intestine with different feeding habits [30, 31]. Liu [32] reported decreased enzyme activity in wild freshwater fishes as compared with farmed fish, as an influence of their trophic level. Our findings are consistent with previous observations; for example, lower amylase and trypsin in activity in the liver was detected in PACM. Interestingly, the activities of digestive enzymes in the midgut and hindgut were higher in PACM than in POCM; this might imply a stronger digestive ability in PACM. In this study, consistency was observed in the distribution of intestinal mucous cells and the activities of digestive enzymes in the different intestine sections, with gradual decreases from foregut to hindgut, although microorganisms will also affect enzymatic activity [33, 34].

The intestinal tract is a complex system that plays a key role not only in digestion, nutrient absorption and osmoregulation, but also in immune homeostasis [2, 35]. The surface area is constantly bombarded by antigens from the diet and the gut microorganisms; while the intestinal tract is one line of defense against pathogens, it is also regarded as a primary portal for pathogenic invasion in fish [36]. The integrity and stability of the function and structure of the intestinal tract are critical for digestion and nutrient absorption. Moreover, the beneficial effects on host health from the commensal microbiota and their fermentation products are well evidenced [37–39]. Wu [40] attributed efficient digestion, especially of cellulose, and the absorption of nutrients in yellow catfish to the intestinal microbiota. In this study, we investigated the abundance of total bacteria, the Firmicutes, the Bacteroidetes, *Bifidobacterium*, *Enterococcus* spp., *Lactobacillus* spp., *A*. *hydrophila*, Enterobacteriaceae and *Streptococcus* spp. in loach in two cultivation modes and during two seasons. The intestinal microbiota always changed with the host fish and ambient environment, and even with the development phase of the loach. In our study, among loach in PACM, several of the bacteria groups presented higher concentrations in summer, such as the Firmicutes, *Lactobacillus* spp., *A*. *hydrophila*, Enterobacteriaceae and *Streptococcus* spp., whereas the concentrations of the Bacteroidetes, *Bifidobacterium* and Enterobacteriaceae were higher in the fall. However, the abundances of the pathogenic bacteria *Enterococcus* spp., *A*. *hydrophila* and *Streptococcus* spp. significantly decreased among loach in PACM in the fall. In general, the intestine of loach does not much develop as the fish grows, and it usually contains fewer microbiota in the early life phase. With the development of the digestive organ, the species composition and quantities of the microflora are gradually enriched, and their population structures progressively stabilize in the fish intestine. Ringø [41] exposed turbot larvae to *Vibrio pelagius* and observed the changes in the intestinal microbiota: the microbiota first increased but then eventually stabilized. In contrast to the observations of Ringø [41], we detected a decline in the abundance of pathogenic gut bacteria among the loach in PACM in the fall; this finding suggests that paddy fields may be the better environment for the growth and health of juvenile loach. However, the data also showed no significant differences in the growth parameters of loach cultured in ponds or in paddy fields [42]. Further study is needed for a more detailed evaluation of differences between the two culture modes. The Firmicutes are a dominant phylum [43] that includes multiple cellulolytic bacteria, which are closely associated with the bioconversion of feeds in the body [44]. The Bacteroidetes are a dominant phylum present in fish [43, 45] and are known to accelerate the catabolism of plant cell walls [46], although the most comprehensive classification studies of these bacteria have been done on land animals.

Because the loach is an omnivorous species, its diet includes algae, grasses, and other plant debris and organic matter found in the sediment [47]. Due to the relationship between the loach’s feeding habits and its microbiota, we can surmise that Bacteroidetes bacteria are a significant presence in the fish’s intestine. Moreover, an increased abundance of the Bacteroidetes might improve the barrier function of the intestinal mucosa, enhancing the host’s immunity [48]. Some *Lactobacillus* and *Bifidobacterium* are recognized as beneficial for intestinal health in fish, and they may be added to the diet as probiotics that improve fish growth and development. Previous studies, especially of *Bifidobacterium*, have focused mainly on land animals and less on aquatic animals. It has also been reported that some lactic-acid bacteria isolated from the gastrointestinal tract of fish can act as probiotics [49, 50]. In addition, *Lactobacillus* can inhibit the growth of Enterobacteriaceae [51] and *Streptococcus* spp. [52]. *Bifidobacterium* is often detected in water as well as in the digestive tracts of fish [53]. Itami [54] found that peptidoglycan derived from *Bifidobacterium thermophilum* enhanced disease resistance in kuruma shrimp. *Bifidobacterium* also can inhibit the growth of *Enterococcus* spp. [55]. Hence, we conclude that it is likely that the pathogenic bacteria might be controlled or even reduced in the presence of probiotics.

*Aeromonas hydrophila* is one of the most common bacteria in freshwater habitats, and it is a frequent cause of disease among cultured and wild fishes worldwide [56]. It is an opportunistic pathogen in both fish and terrestrial animals, including mammals. Consequently, it is important to maintain excellent water parameters for loach in either PACM or POCM. In this study, higher abundances of *A*. *hydrophila* were observed in the intestinal contents and mucosa, for both culture modes, but especially in summer. Fortunately, high abundances in PACM were not maintained during the fall.

Rearing loach in PACM represents a good rice–fish co-culture system [57]. Our observations indicate that particular attention should to be paid to the loach culture management strategy for the summer season. Enhancement of fish immunity is possibly the most promising method for preventing fish diseases; even so, the health condition of freshwater fish is also strongly affected by their trophic level [32]. Therefore, improvements to the feeding strategy for fishes reared in paddy fields needs more attention. The large area of rice in a paddy field might present an obstacle for loach as they swim to feed.

## Conclusions

This study evaluated differences in the digestive enzyme activities of the intestine, the distribution of intestinal mucous cells, and the quantities of some taxa of intestinal microbiota in loach cultured in paddy fields and ponds. The abundance of most bacterial groups in the loach gut presented significant differences between the two cultivation modes, in both summer and fall. However, in both cultivation modes, the pathogenic bacterium *A*. *hydrophila* maintained a relatively high abundance in the intestinal contents and mucosa, including during summer, although its abundance decreased during the fall. This finding indicates that particular attention should to be paid to the loach culture management strategy for the summer season.

## Abbreviation

AB-PAS: Alcian blue and periodic acid schiff
LM: Light microscopy
one-way ANOVA: One-way analysis of variance
PACM: Paddy-cultivated mode
PCR: The polymerase chain reaction
POCM: Pond-cultivated mode
Q-PCR: Quantitative PCR
SD: Standard deviation
